# “It only takes two minutes to ask”—a qualitative study with women on using the FIGO Nutrition Checklist in pregnancy

**DOI:** 10.1002/ijgo.13322

**Published:** 2020-09-07

**Authors:** Sarah Louise Killeen, Shauna L. Callaghan, Chandni Maria Jacob, Mark A. Hanson, Fionnuala M. McAuliffe

**Affiliations:** ^1^ UCD Perinatal Research Centre School of Medicine National Maternity Hospital University College Dublin Dublin Ireland; ^2^ School of Nursing, Midwifery and Health Systems University College Dublin Dublin Ireland; ^3^ Institute of Developmental Sciences University of Southampton Southampton UK; ^4^ NIHR Southampton Biomedical Research Centre University Hospital Southampton Southampton UK

**Keywords:** Acceptability study, Antenatal care, Feasibility study, FIGO Nutrition Checklist, Gestational weight gain, Nutrition, Obesity, Pregnancy, Screening tool

## Abstract

**Objective:**

To gain an in‐depth understanding of how the FIGO Nutrition Checklist could work in clinical practice, from the perspective of pregnant women.

**Methods:**

This qualitative study was part of a pilot study of the FIGO Nutrition Checklist in the antenatal department of a tertiary‐level university maternity hospital in Dublin, Ireland. Individual semistructured phone interviews were conducted with pregnant women who had completed the FIGO Nutrition Checklist as part of the pilot. Interviews were transcribed verbatim and analyzed using content analysis after manual coding of transcripts. Themes and subthemes are described.

**Results:**

Ten interviews were completed. Subthemes related to the FIGO Nutrition Checklist emerged including ease of use and comprehension. Participants discussed how the tool could add value to their appointment by supporting initiation of nutrition conversations and highlighting nutritional issues. The first trimester was identified as the highest priority for using the FIGO Nutrition Checklist. The convenience of having nutrition addressed as part of standard care, rather than a separate appointment, also emerged.

**Conclusion:**

Women in this study had a desire for nutrition and weight to be addressed by clinicians during routine antenatal appointments. The findings support using the FIGO Nutrition Checklist to address this.

## Abstract 2

Pregnant women want to discuss nutrition and weight with their doctors during routine care. The FIGO Nutrition Checklist is an acceptable tool to support this.

## 1 Introduction

Optimal nutrition and weight during pregnancy has the potential to improve maternal and child health and reduce the global burden of noncommunicable diseases.^1^ Many clinical practice guidelines in obstetrics and gynecology therefore recommend routine dietary and weight management counselling for all women.^2^ The aim of this is to meet the nutritional requirements for a healthy pregnancy, manage gestational weight gain, and prevent pregnancy complications.^3^

During pregnancy, women may be more motivated to make diet or lifestyle changes.^4^ Some pregnant women may view diet as one of the factors that are in their control and can help protect their health and the health of their future children.^5^ Previous research has found that pregnant women consider doctors to be the most reliable source for nutrition information in pregnancy and report following the dietary advice provided by clinicians.^6^, ^7^ Despite this, studies demonstrate that women may not receive nutrition advice during pregnancy unless they specifically request it and practices around nutrition advice vary substantially.^8^, ^9^ The effect of this can be seen in the lack of adherence to recommended healthy dietary intakes for pregnancy internationally.^10^

Previous work identified lack of resources and relevant training as barriers to addressing nutrition in practice.^11^, ^12^, ^13^ The FIGO (International Federation of Gynecology and Obstetrics) Nutrition Checklist is designed to facilitate brief and relevant nutrition discussions between women and their healthcare professional, in a personalized and consistent manner. Our survey‐based study evaluating the acceptability and feasibility of the Checklist in clinical practice suggested that women were receptive to discussing nutrition during pregnancy.^14^ In this paper, we discuss the findings of a qualitative study conducted to gain a deeper understanding of how the FIGO Nutrition Checklist could work in practice, from the perspective of pregnant women, some of whom had experience using it as part of a pilot study.^14^

## 2 Materials and methods

This qualitative study is a follow‐on study of a pilot trial of the FIGO Nutrition Checklist that took place in the outpatient department of the National Maternity Hospital, a busy maternity hospital in Dublin, Ireland, between October 7, 2019 and December 12, 2019.^14^ As such, a pragmatic epistemological approach was taken, and a descriptive phenomenological methodology was employed.^15^, ^16^ This is appropriate to understand the lived experiences of women during pregnancy and consider how this applies to the use of the FIGO Nutrition Checklist in the “real‐world” clinical setting.

The phenomenon under investigation was the experiences of women receiving antenatal care in the context of diet and nutrition and the real or hypothesized impact of the FIGO Nutrition Checklist, from the perspective of the woman. Data were analyzed through content analysis to allow for the exploration of data in relation to predefined themes of most interest to the practical application of the findings, in particular, perceptions of the FIGO Nutrition Checklist.^17^ This method was chosen to understand what it is like to receive maternity care in Ireland, as it is experienced by the women, without consideration of varying social, political, or other contexts. Both SLK and SC were involved in the clinical pilot of the FIGO Nutrition Checklist, from which these women were sampled.^14^ Both researchers have professional backgrounds of relevance (registered dietitian and registered midwife), although neither had a clinical relationship with the women they interviewed. Details of the study are reported in accordance with the COREQ checklist for qualitative interview reporting.^18^ Full ethical approval was obtained from the hospital ethics committee (EC202019). Written consent of the participants was obtained. The FIGO Nutrition Checklist is given as supporting information S1.

Data were collected from a purposive sample of participants recruited as part of the pilot study of 125 pregnant women at a tertiary‐level university maternity hospital where maternity care is predominantly obstetrician‐led.^14^ Women of any gestation or age attending routine antenatal clinics were eligible to take part in the pilot. Consent to be contacted for this research was obtained in verbal and written format during the original pilot study. All women interested in the qualitative interviews provided their telephone details on the consent form. Out of this group, women were eligible to take part in the interviews if they were English‐speaking and attended one of the pilot clinics for the study. Telephone interviews were conducted until data saturation was achieved.

A semistructured topic guide with broad and open‐ended questions (Table 1) was created and piloted with members of the wider research team who have extensive experience conducting quantitative and qualitative research with the target group. Before the interview, participants were informed of the interviewer’s background and the reasons for the research. Interview questions were asked with the open‐ended design; however, subsequent prompts and more direct questioning were employed if there was a misunderstanding of questions or further clarification or detail was needed on a particular topic. Interviews were conducted approximately 8 weeks after participation in the pilot study in the antenatal clinic. The qualitative interviews were audio recorded and field notes were created during and after the interviews to supplement the analysis. The duration of the interviews ranged from 11–30 minutes. The research team created a transcription notation system and the audio recordings were transcribed verbatim into anonymized written orthographic transcripts.

**Table 1:** Topic guide for qualitative interview

Theme	Summary of key questions asked
Theme 1. Nutrition, weight, and health	Could you give me an idea of what you think about food and how it effects your health? Now you’re pregnant, are your thoughts different?
Theme 2. Nutrition messages	What type of messages or information have you received about these areas during your pregnancy? Where and how do you get these? What motivates you to seek this information?
Theme 3. Perception of the FIGO Nutrition Checklist in practice	What do you think about the checklist? How did you find talking about the checklist? What is your opinion on using the checklist for all pregnant women?

Qualitative interviews were analyzed using content analysis by SLK and SC.^17^ All 10 transcripts were manually coded line by line independently by each researcher. Themes were predefined based on the questions in the topic guide and, after initial coding, subthemes emerged within these concepts through data analysis (Table 2). Themes were later compared, and agreement was reached through discussion. Transcripts or findings were not returned to participants although clarification of concepts and ideas was obtained during the interview through direct questioning, paraphrasing, and reflection.

**Table 2:** Predefined themes and associated subthemes derived from analysis

Theme	Subthemes
1. Nutrition, weight, and health	Prevention of disease Health and development of baby Pregnancy issues Well‐being
2. Nutrition messages	Mixed/inconsistent Different source of information
3. Perception of the FIGO Nutrition Checklist in practice	
3a. Completing the FIGO Nutrition Checklist	Comprehension
	Time
3b. Value of the FIGO Nutrition Checklist in a clinical setting	Initiate conversations
	Highlight nutritional issues
	Clarify details
	Self‐reflection
	Compare against guidelines
	Motivation for behavior change
3c. Application of the FIGO Nutrition Checklist	Time management
	Part of standard care
	Early pregnancy
	Brief discussion or referral
	Personalized

## 3 Results

### 3.1 Demographics

**Figure 1:**
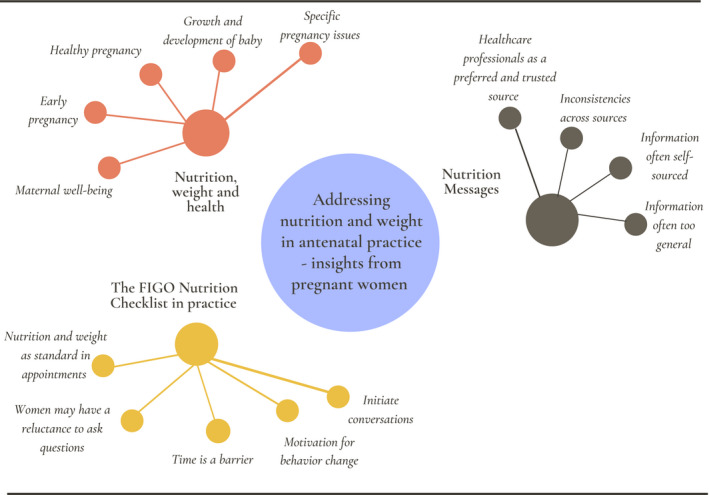
Insights from pregnant women on addressing nutrition and weight in antenatal practice

Of the 10 women who completed an interview, four were in late pregnancy (26–38 weeks of gestation) and six were 1–2 months postnatal having completed the FIGO Nutrition Checklist study in late pregnancy. Of the 10 participants, six were having their first baby. The subthemes that emerged from analysis of the interviews are given in Table 2 and outlined in Figure 1.

### 3.2 Nutrition, weight, and health

There was a clear perceived importance of nutrition and weight for maternal health among participants. Maternal benefits discussed included the prevention of disease and enhanced well‐being. Women attributed greater importance to these factors during pregnancy as they highlighted the links to improved child health outcomes. The important role of nutrition in preventing and managing pregnancy‐specific issues, such as anemia and gestational diabetes, was noted and other issues, such as cravings and nausea, were identified as barriers to healthy eating during pregnancy.

*P9: Good food is critical for good health…I think it gives good energy and that it keeps you fit.*

*P5: I suppose like first few months I was a bit nauseous so you know there was like certain foods that…I couldn't really eat…I probably could've eaten better…if it wasn't for the cravings.*

### 3.3 Nutrition messages

When discussing their experiences of receiving nutrition advice for pregnancy to date, several themes emerged including limited experiences with healthcare professionals and identifying conflicting messages when searching for advice from other sources. It was highlighted that in the absence of these nutrition‐related issues, women received little nutrition information. In addition, the advice received as part of antenatal classes was considered impersonal with insufficient follow‐up.

*P2: I don’t think in the antenatal clinic there was a specific emphasis on what I’m eating…I suppose they were very kind of keen to let me know you should keep up your iron levels…but there was no real follow‐up to that in my opinion.*

*P7: After I got pregnant I was a bit confused as like, do I eat this, do I not eat that, who do I ask or what do I do, and people around you give you different opinions so I wasn't sure.*

### 3.4 Perception of the FIGO Nutrition Checklist

Within the interview, participants were asked to reflect on their initial impressions of the FIGO Nutrition Checklist and their perceptions of incorporating it into routine antenatal care. Themes that emerged from this discussion included the ease of use of the FIGO Nutrition Checklist, with all women stating it was clear, simple, and quick to complete.

*P2: I think it was very easy to understand and very easy to give a view to an extent.*

#### 3.4.1 Value of resource

The added value of the FIGO Nutrition Checklist as a resource also emerged as a theme, with women stating it provided good information, highlighted potential issues, and clarified some misconceptions about nutrition in pregnancy. Women found it could be used to initiate conversations and could act as a reminder to ask questions. Using the FIGO Nutrition Checklist was identified as a facilitator of behavior change as it enabled reflection on current behaviors.

*P8: …having to actually physically answer the questions made me like very much aware of what I was doing.*

*P9: So if the doctor had like 5 10 minutes and filled it out I think it's a good idea…if you're left with the questionnaire alone…probably not.*

#### 3.4.2 Routine application of the checklist

All women welcomed the suggestion of incorporating a tool such as the FIGO Nutrition Checklist into a routine part of antenatal care as a means for facilitating brief discussions. They acknowledged that women could be referred to a dietitian for more in‐depth assessment if required. Participants outlined that addressing nutrition in this brief format would save women time rather than having to come to a separate appointment with a dietitian or other professional. The limited time allocated for face‐to‐face interaction with healthcare professionals emerged, in particular the need to discuss other concerns or aspects of pregnancy were highlighted by most women as the reason for not discussing their responses on the FIGO Nutrition Checklist during their appointment. Participants perceived the FIGO Nutrition Checklist to be best placed as a tool for early pregnancy, especially for first‐time mothers. Some women thought that the FIGO Nutrition Checklist could be used in all appointments and adapted as appropriate.

*P7: …it will be good to include diet and nutrition into the routine assessment or else every clinic appointment.*

*P6: …it only takes two minutes to ask someone have they got any dietary questions.*

## 4 Discussion

Like previous studies, we found that women do not receive adequate or personalized nutrition advice in pregnancy and that it is limited to addressing specific pregnancy issues such as anemia and food safety.^19^, ^20^ This may have a negative effect on behavior change as previous studies have found women may be less likely to follow generic advice.^21^ In our study, we found that women reported confusion regarding appropriate diet for pregnancy and, worryingly, they felt they could not ask their doctor for clarification. Mistaken or false beliefs are considered a barrier to women meeting dietary requirements for pregnancy.^7^ We found women had a desire for nutrition to be addressed as “part of the process” in antenatal care. The women hypothesized many potential benefits to this including frequent reminders of the need for a healthy diet and a chance for periodic reflection, in a convenient manner as part of their standard appointment. A study by Bookari et al.^22^ found that women had a desire to address nutrition concerns in their usual antenatal appointment and were frustrated when referred elsewhere as referral was associated with long wait times.

Many pregnant women do not follow dietary advice for pregnancy and, as part of this work, we found that most women reported at least one undesirable dietary practice during pregnancy.^10^, ^14^ The findings of our study confirm extensive previous research showing that pregnant women welcome diet, weight, and nutrition‐related discussions.^8^, ^23^ Clinicians working with pregnant women have a responsibility to incorporate brief nutrition and weight discussions into their clinical appointments. Despite this, multiple systematic reviews have found that the lack of prioritization of behavior change interventions by healthcare professionals is a barrier to incorporating it into practice.^11^ In a national survey in the UK completed in 2017, 50% of healthcare professionals said that they did not provide patients with opportunistic behavior change techniques even though they perceived there was a need to do so.^24^ Other barriers such as lack of time, competing priorities during pregnancy assessments, and lack of training and resources have been reported by healthcare professionals.^11^, ^13^ In the same UK survey, providing behavior change interventions took 35.3% of the appointment time.^24^ However, the women in our study considered the FIGO Nutrition Checklist to be a quick intervention that is appropriate for use within a typical antenatal appointment. As the women in this study completed the data collection aspect of the FIGO Nutrition Checklist in advance of face‐to‐face clinic time, using the tool in this way, if appropriate to the clinical setting, may be useful to enhance efficiency in the process. While localizing and implementing the FIGO Nutrition Checklist in the future, support for clinicians needs to be provided in addition to a contextualized version of the checklist.^25^

Understanding the needs and opinions of service users is essential to providing quality care. To the best of our knowledge, this is the first qualitative study to investigate a simple, free, clinical practice tool specifically designed for obstetricians and gynecologists as a potential solution to initiate conversations related to general healthy nutrition and weight in antenatal care. The strengths of this study include the in‐depth analysis of the value of nutrition in pregnancy and how the FIGO Nutrition Checklist can work in clinical practice from the woman’s perspective. The rich data collected from participants, who are service users, may serve to inform the implementation of the FIGO Nutrition Checklist in a clinic setting and provide justification for use.

There are a few limitations that are worth noting. This was a single‐center study with English‐speaking women from general antenatal clinics. We did not collect data on ethnicity, socioeconomic status, or medical history other than parity. It is therefore unclear how the findings of this study will translate to other clinical situations and locations. Future work could employ varied purposive sampling to explore the influence of these considerations. Despite this, the study was well designed to answer the key research question of how the FIGO Nutrition Checklist could work in practice from the perspective of pregnant women in general and provides justification for further research and use in practice. A key area for future work would be to explore the attitudes of obstetricians and gynecologists to using the FIGO Nutrition Checklist, including exploring the issue of time constraints that may act as a barrier to using the Checklist in practice.

In conclusion, pregnant women appear to have a strong preference for nutrition to be addressed by healthcare professionals, during routine antenatal care. A myriad of positive themes relating to the FIGO Nutrition Checklist emerged from the interviews that encourage the use of the tool as a solution to the reported deficits in terms of nutritional care in standard clinic appointments during pregnancy.

## Author Contributions

All authors were involved in the conception and design of the study. SLK and SC conducted the pilot study and all aspects of data collection and analysis. SLK wrote the manuscript with input from all other authors. All authors provided input into the study design, analytical methods, and revisions of the manuscript.

## Conflicts of Interest

The authors have no conflicts of interest.

## Supporting information


**Supporting information S1**. FIGO nutrition checklist for pre‐pregnant/early pregnant women. Reproduced with permission from FIGO.
